# Prediction of sarcopenia using a battery of circulating biomarkers

**DOI:** 10.1038/s41598-021-87974-6

**Published:** 2021-04-21

**Authors:** Rizwan Qaisar, Asima Karim, Tahir Muhammad, Islam Shah, Javaidullah Khan

**Affiliations:** 1 Basic Medical Sciences, College of Medicine, University of Sharjah, Sharjah, United Arab Emirates; 2 University of Health Sciences, Lahore, Pakistan; 3 Departmenr of Biochemistry, Gomal Medical College, Dera Ismail Khan, Pakistan; 4 Department of Cardiology, Al Qassimi Hospital, Sharjah, United Arab Emirates; 5 Department of Cardiology, Post Graduate Medical Institute, Hayatabad Medical Complex, Peshawar, Pakistan

**Keywords:** Biomarkers, Cardiology, Diseases, Medical research, Molecular medicine

## Abstract

Loss of muscle mass and strength with aging, termed sarcopenia is accelerated in several comorbidities including chronic heart failure (CHF) and chronic obstructive pulmonary diseases (COPD). However, the effective circulating biomarkers to accurately diagnose and assess sarcopenia are not known. We recruited male healthy controls and patients with CHF and COPD (n = 81–87/group), aged 55–74 years. Sarcopenia was clinically identified based on hand-grip strength, appendicular skeletal muscle index and physical capacity as recommended by the European working group for sarcopenia. The serum levels of amino-terminal pro-peptide of type-III procollagen, c-terminal agrin fragment-22, osteonectin, irisin, fatty acid-binding protein-3 and macrophage migration inhibitory factor were significantly different between healthy controls and patients with CHF and COPD. Risk scores for individual biomarkers were calculated by logistic regressions and combined into a cumulative risk score. The median cutoff value of 3.86 was used to divide subjects into high- and low-risk groups for sarcopenia with the area under the curve of 0.793 (95% CI = 0.738–0.845, *p* < 0.001). A significantly higher incidence of clinical sarcopenia was found in high-risk group. Taken together, the battery of biomarkers can be an effective tool in the early diagnosis and assessment of sarcopenia.

## Introduction

Sarcopenia is traditionally defined as the loss of muscle mass with aging^1^. However, recent definitions of sarcopenia consider the loss of muscle mass, strength and physical capacity with aging^2^. For example, the European Working Group for Sarcopenia (EWGSOP) defines sarcopenia as muscle atrophy (normalized for height) combined with muscle weakness (measured in hand-grip muscles) and/or reduced physical capacity (measured via gait speed)^3^. Among these parameters, muscle weakness has emerged as the primary determinant of sarcopenia and functional dependency in aging^2^. Elderly with low muscle mass (normalized for height) in the absence of muscle weakness and functional compromise are categorized in “pre-sarcopenia” phase, while “probable sarcopenia” is defined as reduced hand-grip strength (HGS) and/or compromised physical capacity (low chair stand test ability)^2^, which warrants further assessments. These assessments highlight the importance of sarcopenia as the muscle impairment in the elderly is often associated with a dependent lifestyle due to physical decline^4^, falls^5^, fractures^5^ and increased morbidities^6^.

Several methods are used to assess the indexes of sarcopenia. MRI and CT scans are considered the gold standards to measure muscle mass but are costly, require technical expertise and involve radiation exposure. Bioelectrical impedance is now widely implied due to its simple and cost-effective manner despite less reliability due to gender, ethnicity and hydration status^7^. Muscle strength is routinely measured in the hand-grip muscles with a dynamometer and shows a correlation with several age-related diseases^8–10^. Additionally, a short physical performance battery (SPPB) has emerged as a promising tool to evaluate functional capacity and incorporates standing balance and chair stand test in addition to gait speed^11^. SPPB has shown high sensitivity for sarcopenia and maybe a favorable tool in clinical settings as it is a simple and quick test, which does not require any specialized instruments.

In addition to clinical measurements, sarcopenia is also definable by simple functional questions. SARC-F questionnaire has emerged as a useful tool for the rapid assessment of sarcopenia^12^. It considers various measures of functional independence such as strength, assistance in walking, rising from a chair, climbing stairs and falls. Each component is awarded a score from 0 to 10, with a maximal score of 10 in the overall assessment. A score ≥ 4 is considered as a predictor of sarcopenia^12^.

Several co-morbidities can induce and/or exacerbate the sarcopenia phenotype in aging. These diseases also share several common risk factors and health outcomes with sarcopenia. However, sarcopenia is frequently underdiagnosed in clinical practice despite the considerable overlap in the severities of sarcopenia phenotype and comorbidities. Further, treatment protocols for sarcopenia do not take comorbidities into account^13^. Therefore, there is a need to determine the diagnosis and prevalence of sarcopenia in the elderly with age-related comorbidities. Among them, chronic obstructive pulmonary diseases (COPD) and congestive heart failure (CHF) are common drivers of sarcopenia in the elderly^10,14^. We have previously shown that patients with respiratory diseases have accelerated loss of muscle mass and strength than the age-matched healthy elderly^9^.

These diseases also share several common risk factors and health outcomes with sarcopenia. However, sarcopenia is frequently underdiagnosed in clinical practice despite the considerable overlap in the severities of sarcopenia phenotype and comorbidities. Further, treatment protocols for sarcopenia do not take comorbidities into account^13^. Therefore, there is a need to determine the diagnosis and prevalence of sarcopenia in the elderly with comorbidities such as COPD and CHF.

Several molecular mechanisms contribute to the pathogenesis of sarcopenia. Both the intrinsic (generalized inflammation, oxidative stress, calcium dysregulation, apoptosis, hypoxia, the disintegration of neuromuscular junction) and extrinsic (chronic inactivity, poor nutrition, endocrine dysfunction) factors contribute to the loss of muscle mass and strength in aging^15^. However, considering the complex pathophysiology, no single molecular biomarker can accurately evaluate the sarcopenia phenotype, necessitating the use of multiple biomarkers to assess loss of muscle mass and strength in the elderly.

Given the complexity of sarcopenia, we tested a battery of circulating biomarkers as a strategy to define a biomarker panel specific to different pathophysiological mechanisms of sarcopenia. We evaluated specific biomarkers to assess the neuromuscular junction integrity (c-terminal agrin fragment 22 or CAF22), protein turnaround (amino-terminal pro-peptide of type III procollagen or P3NP), cell–matrix interaction (osteonectin), growth factors (irisin), cellular metabolism (fatty acid-binding protein 3 or FABP3) and systemic inflammation (macrophage migration inhibitory factor or MIF). The circulating biomarker levels were correlated with the sarcopenia as defined by clinical parameters or SARC-F questionnaire to assess their diagnostic significance. The comparative analysis of the biomarkers was performed in the normal elderly with sarcopenia and the patients of COPD and CHF with advanced sarcopenia.

## Results

### Characteristics of the participants

The basic characteristics of the study population are summarized in Table 1.

**Table 1:** Body composition, physical parameters and circulating biomarkers in healthy controls and patients with COPD and CHF.

	Healthy	COPD	CHF
**Age at baseline (years)**	62.6 ± 5.5	64.3 ± 3.7	66.9 ± 5.4
**Body composition**			
BMI (Kg/m^2^)	23.85 ± 2.76	22.27 ± 3.25	25.62 ± 3.32*#
ASM (Kg)	20.29 ± 3.08	19.27 ± 2.61	19.41 ± 3.24
ASMI (Kg/m^2^)	7.24 ± 1.45	7.03 ± 1.38*	6.84 ± 1.44*
Percent fat	27.79 ± 3.15	25.18 ± 3.85*	28.43 ± 3.63#
**Physical parameters**			
HGS (kg)	25.57 ± 5.36	20.19 ± 4.37*	21.45 ± 3.31*
HGS / ASM	1.26 ± 0.18	1.04 ± 0.14*	1.01 ± 0.17*
Daily steps count	6873 ± 1394	3182 ± 949*	3648 ± 1203*
4-meter gait speed (m/s)	1.23 ± 0.21	0.87 ± 0.18*	0.83 ± 0.17*
Chair stand for five rises (s)	13.5 ± 2.54	15.4 ± 3.11	16.7 ± 3.69*
**Spirometry and oxygen saturation**			
FEV1% predicted	97.39 ± 3.3	61.89 ± 5.7*	66.31 ± 4.3*#
PEFR % predicted	93.53 ± 4.7	67.39 ± 4.5*	73.61 ± 4.1*
SpO_2_	98.5 ± 1.5	95.3 ± 2.2	95.1 ± 2.4
**Plasma biomarkers**			
Plasma 8-isoprostanes (pg/ml)	58.4 ± 12.7	95.2 ± 16.9*	87.9 ± 13.4*
CRP (mg/l)	1.91 ± 0.24	3.26 ± 0.43*	3.53 ± 0.47*#
Creatine kinase (IU/L)	167.9 ± 25.7	293.5 ± 52.7*	279.9 ± 42.9*

Values are expressed as mean ± SD, one-way analysis of variance. *p < 0.05 *vs.* healthy controls; #*p* < 0.05 *vs.* COPD participants; (N = 83–88/group). (BMI; body mass index, ASM; appendicular skeletal mass, ASMI; appendicular skeletal mass index, HGS; hand-grip strength, FEV1; forced expiratory volume in 1 s, PEFR; peak expiratory flow rate, CRP; C—reactive protein).

**Figure 1:**
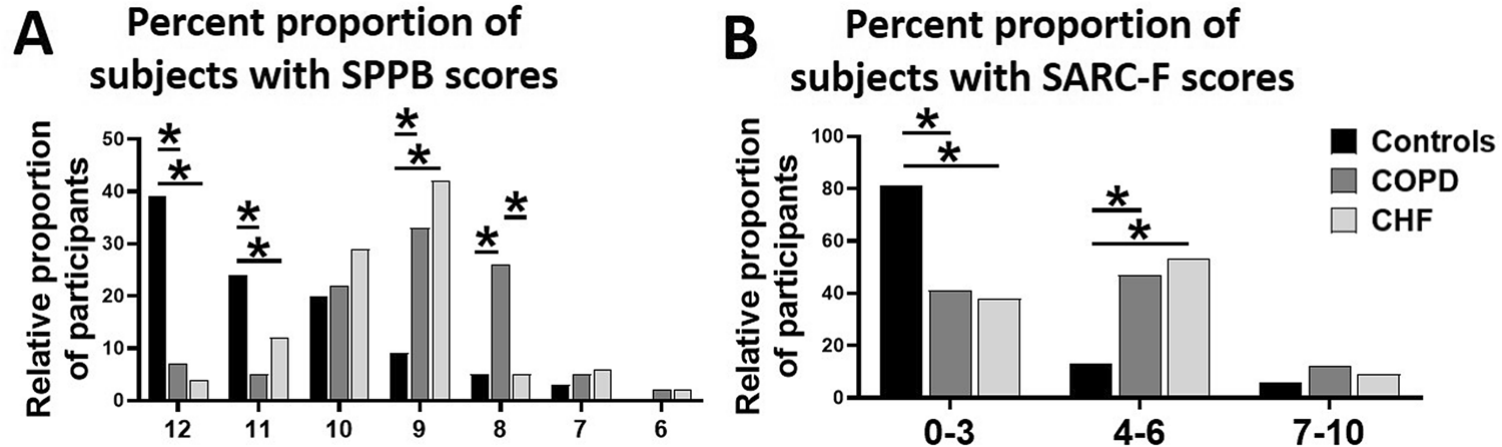
Relative proportions of the participants with different SPPB (**A**) and SARC-F (**B**) scores in the healthy controls (N = 87) and patients with COPD (N = 86) and CHF (N = 81). Values are expressed in percentages, *p < 0.05.

Overall, the COPD and CHF patients had lower ASMI, HGS, gait speed and steps count than healthy controls (all *p* < 0.05). Moreover, the circulating levels of 8-isoprostanes, CRP and CK were higher in the patients with COPD and CHF than healthy controls (all *p* < 0.05). These patients also performed poorly on the SPPB and SARC-F scales. Generally, a higher score means a good functional capacity on SPPB scale, but poor functional capacity on SARC-F scale. Thus, the relative proportion of participants with SPPB score ≤ 8 (among the diagnostic indexes of sarcopenia) was higher in patients with COPD and CHF than healthy controls (≈64% and ≈68% *vs.* ≈9%, respectively, *p* < 0.05) (Fig. 1A). We next used the SARC-F questionnaire as a measure of functional dependence to assess sarcopenia. A cut-off score of ≥ 4 was chosen as the diagnosis of sarcopenia^12^. In agreement with the alterations in the SPPB score, a significantly higher incidence of sarcopenia was found in the patients with COPD and CHF than healthy controls (≈57% and ≈63% *vs.* ≈21%, respectively, *p* < 0.05) (Fig. 1B).

### Circulating biomarkers accurately identify advanced sarcopenia in COPD and CHF.

**Figure 2:**
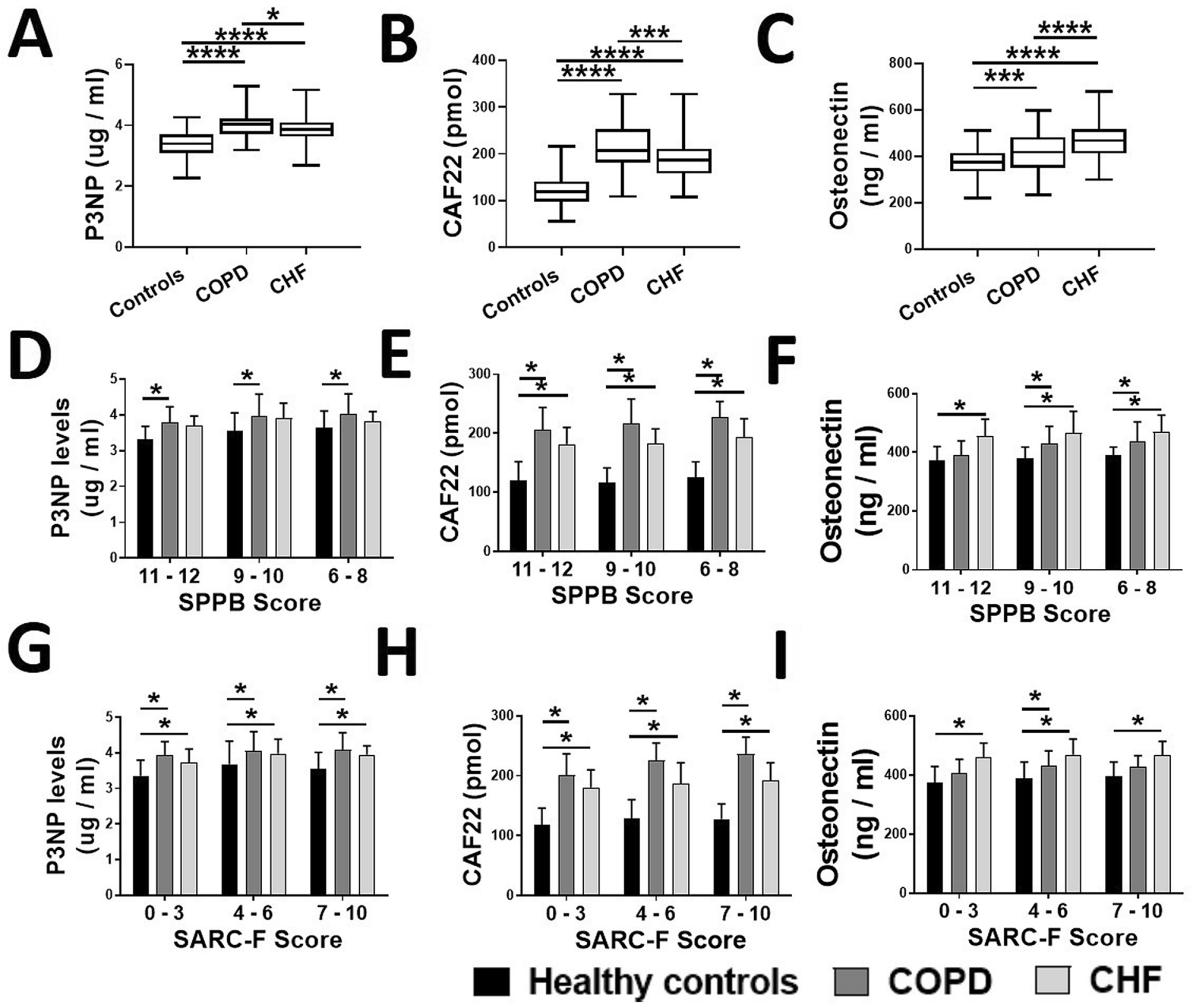
Comparison of circulating P3NP (**A**), CAF22 (**B**) and osteonectin (**C**) levels in healthy controls (N = 87) and patients with COPD (N = 86) and CHF (N = 81). The biomarkers levels were generally higher in the participants with advanced sarcopenia based on SPPB (**D**, **E**, and **F**) and SARC-F (**G**, **H**, and **I**) scoring. Values are expressed as mean ± SD, one-way analysis of variance. *p < 0.05.

**Figure 3:**
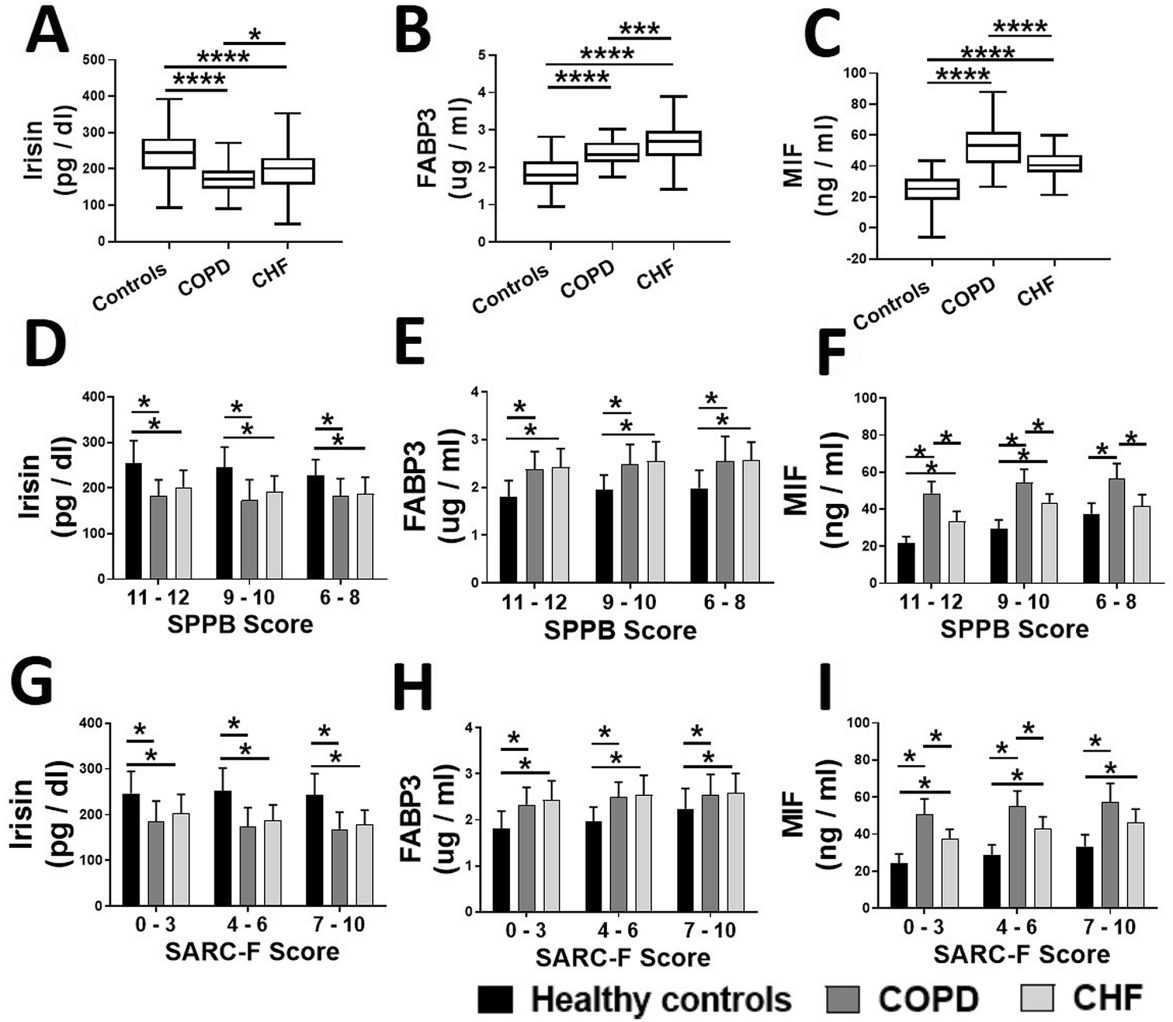
Comparison of circulating irisin (**A**), FABP3 (**B**) and MIF (**C**) levels in healthy controls (N = 87) and patients with COPD (N = 86) and CHF (N = 81). The biomarkers levels were generally higher in the participants with advanced sarcopenia based on SPPB (**D**, **E**, and **F**) and SARC-F (**G**, **H**, and **I**) scoring. Values are expressed as mean ± SD, one-way analysis of variance. *p < 0.05.

Candidate biomarkers for sarcopenia were selected based on the relevant literature and our previous investigations in patients with normal and advanced phases of sarcopenia^9,10,16^. Among various candidates investigated, circulating levels of P3NP, CAF22, osteonectin, irisin, FABP3 and MIF were significantly different in the COPF and CHF subjects with advanced sarcopenia, when compared to age-matched healthy controls. Circulating P3NP, CAF22, osteonectin, FABP3 and MIF levels were higher while irisin levels were lower in COPD and CHF patients than healthy controls (all *p* < 0.05) (Figs. 2A–C, 3A–C). We also analyzed the levels of circulating biomarkers according to SPPB and SARC-F categorization. Significant variations were found in the biomarker levels according to the disease status and categorizations of the SPPB = and SARC-F scales (Figs. 2D–I, 3D–I). In general, the categories with sarcopenia status (SPPB score ≤ 8 or SARC-F score ≥ 4) were associated with higher levels of P3NP, CAF22, osteonectin, FABP3 and MIF and lower levels of irisin in the individual cohorts of healthy controls, COPD and CHF patients.

### Application of predictive probabilities of sarcopenia using combinations of biomarkers

**Figure 4:**
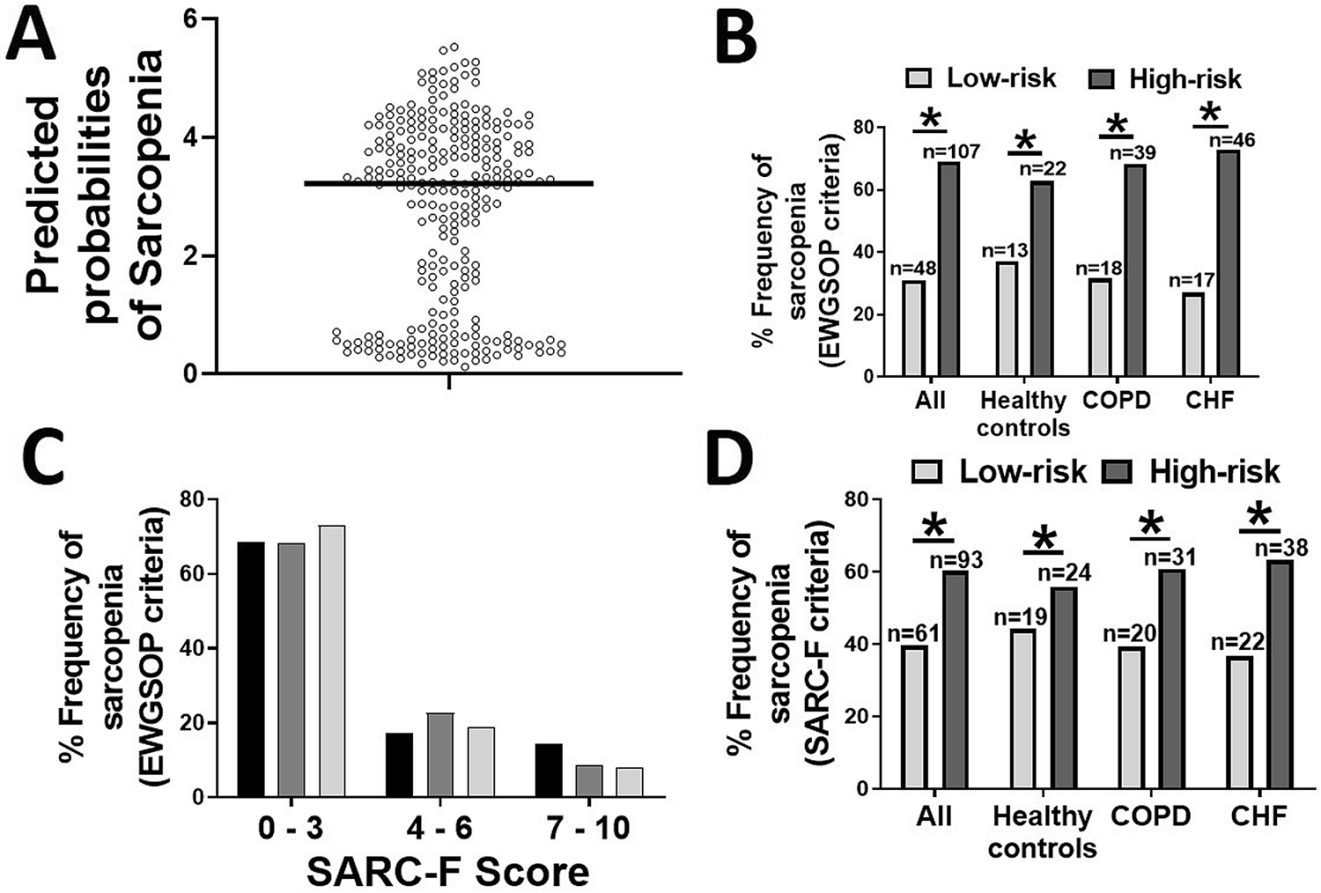
Cumulative risk score for all the participants based on six biomarkers (P3NP, CAF22, osteonectin, irisin, FABP3 and MIF). The scatter plot of the participants with the median risk score (cutoff value = 3.86) was applied to divide into high- and low-risk groups (**A**). The relative proportion of the clinically diagnosed sarcopenic patients in the two risk-groups (**B**) and participants’ categorization based on SARC-F scores (**C**) in the three study cohorts. The relative proportion of the sarcopenic patients as defined by SARC-F criteria in the two risk-groups (**D**) in healthy controls and patients with COPD and CHF. *p < 0.05.

Since individual biomarkers often lack in specificity and/or sensitivity in the assessment of sarcopenia than the panels of biomarkers, we next integrated the measurements of several biomarkers to enhance the diagnostic potential of sarcopenia. We first generated predicted probabilities of sarcopenia using logistic regression coefficients of individual biomarkers. We then added the predicted probabilities of all biomarkers to generate risk scores for each individual participant. All participants were divided into the high-risk and low-risk groups based on the median cut-off value of 3.86 (Fig. 4A). We found a significantly higher proportion of sarcopenia patients (based on EWGSOP criteria) in the high-risk than low-risk groups (*p* < 0.05; 95% CI of diff. − 59.05 to − 14.28), indicating the high diagnostic potential of the biomarkers panel (Fig. 4B). A similar trend was found in individual cohorts of healthy controls, COPD and CHF participants, who had significantly higher proportions of sarcopenia subjects in the high-risk groups. These findings indicate the diagnostic potential of the panel of biomarkers irrespective of disease status. We next evaluated the efficacy of SARC-F in diagnosing sarcopenia by evaluating the relative proportions of clinically sarcopenic patients for each category of SARC-F questionnaire. ≈70% of clinically diagnosed sarcopenic patients scored ≤ 3 on SARC-F, indicating the usefulness of SARC-F in the diagnosis of sarcopenia (Fig. 4C). We next asked whether the biomarkers panel shows a correlation with sarcopenia defined by SARC-F criteria. A significantly higher proportion of sarcopenia patients were found in the high-risk as compared to low-risk groups in all cohorts (*p* < 0.05; 95% CI of diff. − 30.11 to − 10.20) (Fig. 4D) indicating the efficacy of biomarkers panel in diagnosis of sarcopenia.

### Significance of the biomarkers panel in accurate diagnosis of sarcopenia

**Figure 5:**
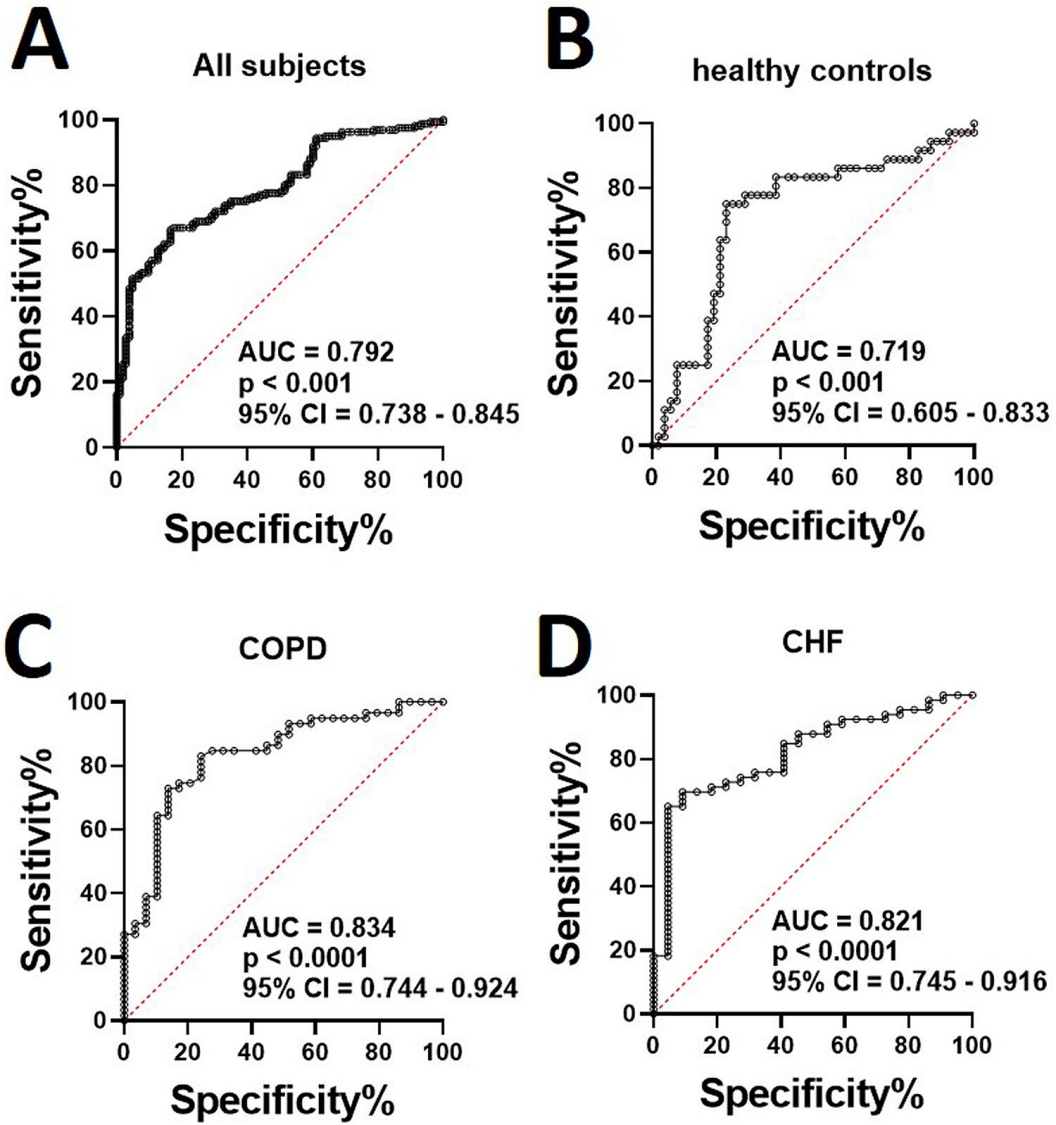
Significance of cumulative risk score for all the participants based on six biomarkers (P3NP, CAF22, osteonectin, irisin, FABP3 and MIF). Receiver operating characteristic (ROC) curves for all the participants (**A**), healthy controls (**B**) and the patients with COPD (**C**) and CHF (**D**). The area under the curve (AUC) was calculated for each group to determine the significance of the biomarkers panel in diagnosis of sarcopenia.

To assess the usefulness of the risk scores for the diagnosis of sarcopenia, we generated ROC curves and measured the sensitivity and specificity of the biomarkers panel for all cohorts of participants (Fig. 5). We obtained significantly high ROC curves for all study groups (AUC > 0.7, all *p* < 0.001). AUC was 0.792 (*p* < 0.001) for all the subjects pooled together (Fig. 5A). Slightly lower AUC was found for the healthy controls (AUC = 0.719, *p* < 0.001) than the diseased groups (Fig. 5B). The AUC values were higher for the patients with COPD (AUC = 0.834) and CHF (AUC = 0.821) indicating the usefulness of the biomarker panel in predicting sarcopenia associated with age-related diseases (Fig. 5C,D). Taken together, these results suggest that the panel of multiple biomarkers can be a useful tool to predict varying degrees of sarcopenia in the healthy and diseased elderly participants.

### Correlation of biomarkers with functional capacity and sarcopenia indexes

We next evaluated the correlation of the circulating biomarkers with the indexes of sarcopenia as defined by the European working group on sarcopenia in older people^17^. All six biomarkers showed varying degrees of correlations with the ASMI, HGS and the gait speed (Table 2). Among the individual biomarkers, CAF22 showed the strongest correlation with ASMI (r^2^ = 0.315, *p* < 0.001) followed by osteonectin (r^2^ = 0.278, *p* < 0.001) and irisin (r^2^ = 0.263, *p* < 0.001) in all subjects. HGS was significantly correlated with all six biomarkers, and the strongest correlation was found with irisin (r^2^ = 0.475, *p* < 0.001). Association of gait speed with the levels of biomarkers was less robust and was the strongest with osteonectin (r^2^ = 0.236, *p* < 0.001). Generally, we found stronger associations between biomarkers levels and the sarcopenia indexes in the COPD and CHF groups than the healthy controls. Irisin and osteonectin emerged as the useful biomarkers to predict sarcopenia indexes in COPD and CHF patients, while the other biomarkers showed varying degrees of correlations with sarcopenia indexes (Table 2).

**Table 2:** Correlations coefficients of circulating biomarkers with indexes of sarcopenia including ASMI, HGS and gait speed in healthy controls and patients with COPD and CHF (N = 83–87 per group).

		**All subjects**	**Healthy controls**	**COPD**	**CHF**
**P3NP**	**ASMI**	− 0.235**	− 0.115*	− 0.099*	− 0.126*
	**HGS**	− 0.197**	− 0.071	− 0.168*	− 0.081
	**Gait speed**	− 0.134*	− 0.094*	− 0.083	− 0.087
**CAF22**	**ASMI**	− 0.315**	− 0.117*	− 0.212**	− 0.246**
	**HGS**	− 0.273**	− 0.149*	− 0.184*	− 0.237*
	**Gait speed**	− 0.192*	− 0.103*	− 0.138*	− 0.127*
**Osteonectin**	**ASMI**	− 0.278**	− 0.132*	− 0.179*	− 0.305**
	**HGS**	− 0.351**	− 0.101*	− 0.296*	− 0.318**
	**Gait speed**	− 0.236**	− 0.153*	− 0.142*	− 0.184*
**Irisin**	**ASMI**	0.263**	0.166*	0.173*	0.218**
	**HGS**	0.475**	0.259*	0.336**	0.506**
	**Gait speed**	0.223**	0.132*	0.297**	0.104*
**FABP3**	**ASMI**	− 0.173*	− 0.048	− 0.128*	− 0.111*
	**HGS**	− 0.241**	− 0.177*	− 0.238**	− 0.126**
	**Gait speed**	− 0.216**	− 0.073	− 0.105*	− 0.195**
**MIF**	**ASMI**	− 0.154*	− 0.123*	− 0.085*	− 0.116*
	**HGS**	− 0.352**	− 0.209**	− 0.321**	− 0.273**
	**Gait speed**	− 0.247**	− 0.168*	− 0.173*	− 0.208**

*p < 0.05, **p < 0.001. (P3NP; amino-terminal of type III pro-collagen peptide, CAF22; C-terminal Agrin fragment-22, FABP3; fatty acid-binding protein 3, MIF; Macrophage migration inhibitory factor).

### Correlation of circulating biomarkers with markers of inflammation, oxidative stress and muscle damage

Since the inflammation, oxidative stress and muscle structural integrity can affect the sarcopenia status, we next evaluated the correlations of the plasma 8-isoprostanes, CRP and CK levels with the selected panel of biomarkers. Plasma 8-isoprostanes levels, a marker of oxidative stress^18^ showed a significant correlation with all the selected biomarkers, with irisin (r^2^ = 0.293), MIF (r^2^ = 0.275) and osteonectin (r^2^ = 0.271) showing the strongest correlation (Table 3). Plasma CRP level, a marker of generalized inflammation showed a significant correlation with all the selected biomarkers when all subjects were pooled together. The strongest correlation was found with plasma irisin (r^2^ = 0.249) and FABP3 (r^2^ = 0.201) levels (Table 3). Circulating CK levels are a marker of muscle damage^19^ and showed the strongest correlations with CAF22 (r^2^ = 0.229) and osteonectin (r^2^ = 0.219) among the selected panel of biomarkers. In all analysis, the values of correlation coefficients were slightly higher in the patients with COPD and CHF, when compared to healthy controls (Table 3).

**Table 3:** Correlations coefficients of circulating biomarkers with plasma 8-isoprostanes, CRP and creatine kinase levels in healthy controls and patients with COPD and CHF (N = 83–87 per group).

		All subjects	Healthy controls	COPD	CHF
**P3NP**	**8-isoprostanes**	0.125*	0.085	0.058	0.082
	**Plasma CRP**	0.119*	0.127*	0.095	0.103
	**Creatine kinase**	0.125*	0.075	0.091	0.124*
**CAF22**	**8-isoprostanes**	0.136*	0.086	0.105	0.128
	**Plasma CRP**	0.157*	0.107	0.139*	0.121*
	**Creatine kinase**	0.229**	0.136*	0.213**	0.286**
**Osteonectin**	**8-isoprostanes**	0.271**	0.131*	0.181*	0.228**
	**Plasma CRP**	0.197**	0.126*	0.149*	0.188**
	**Creatine kinase**	0.219**	0.149*	0.137*	0.178*
**Irisin**	**8-isoprostanes**	− 0.293**	− 0.195**	− 0.238**	− 0.229**
	**Plasma CRP**	− 0.249**	− 0.157*	− 0.169*	− 0.209**
	**Creatine kinase**	− 0.157*	− 0.081	− 0.106*	− 0.117*
**FABP3**	**8-isoprostanes**	0.213**	0.193**	0.183*	0.208*
	**Plasma CRP**	0.201**	0.127*	0.135*	0.174**
	**Creatine kinase**	0.166*	0.143*	0.179*	0.149*
**MIF**	**8-isoprostanes**	0.275**	0.217**	0.241**	0.193**
	**Plasma CRP**	0.173*	0.143*	0.151*	0.126*
	**Creatine kinase**	0.193*	0.154*	0.166*	0.187**

*p < 0.05, **p < 0.001. (P3NP; amino-terminal of type III pro-collagen peptide, CAF22; C-terminal Agrin fragment-22, FABP3; fatty acid-binding protein 3, MIF; Macrophage migration inhibitory factor).

Since inflammation and oxidative stress can contribute to loss of muscle mass and strength in aging^20^, we next measured the circulating markers of inflammation and oxidative stress. We found higher mRNA levels of pro-inflammatory cytokines interleukin-10 (IL-10), transforming growth factor-beta 1 (TGF-β1), C–C motif chemokine receptor 5 (CCR5), (interleukin-8) IL-8 and adrenomedullin (ADM) in patients with COPD and CHF (all *p* < 0.05), indicating increased inflammation in these diseases (supplementary Fig. 1). Additionally, plasma levels of chemokine ligand 2 (CXCL2) were higher in CHF patients. Among the markers of oxidative stress, we found higher expressions of superoxide dismutase 1 (SOD1), catalase, glutathione synthetase (GSS) and glutathione peroxidase 1 (GPX1) in both disease groups than healthy controls (all *p* < 0.05), indicating increased oxidative stress (supplementary Fig. 1).

## Discussion

We investigated the diagnostic potential of six circulating biomarkers related to distinct pathophysiological mechanisms of sarcopenia. Healthy controls and the patients with comorbidities were selected to obtain a wide range of sarcopenia phenotypes from early to advanced stages. A significantly higher incidence of sarcopenia was found in patients with COPD and CHF compared to healthy controls. While the candidate biomarkers were useful in the identification of sarcopenia, the combination of biomarkers enhanced the diagnostic accuracy of sarcopenia in the elderly.

There has been an increasing emphasis to identify the biomarkers of sarcopenia. However, most studies mainly focus on muscle mass and/or strength with relatively little emphasis on the functional capacity in the elderly. Considering that the EWGSOP2 recently updated its definition of sarcopenia^2^, our study better contributes to the biomarker assessment of sarcopenia by evaluating SPPB in the elderly. Additionally, the incorporation of the SARC-F questionnaire in biomarkers evaluation can be useful in assessing early asymptomatic cases of sarcopenia. Given the multifactorial nature of sarcopenia, we selected biomarkers that are evidently correlated with skeletal muscle metabolism, growth, regeneration, and systemic inflammation.

The detonation of the neuromuscular junction (NMJ) is one of the hallmarks of sarcopenia^21^. Agrin is a major protein maintaining NMJ integrity by aggregating the acetylcholine receptors at the post-synaptic terminal. Sarcopenia and other catabolic conditions disrupt NMJ integrity via proteolytic cleavage of agrin into CAF22 that can be detected in circulation. We have previously shown that plasma CAF22 levels are increased in sarcopenia with pulmonary diseases^9,16^. Here, we confirm and extend these findings to sarcopenia with CHF. Patients with accelerated sarcopenia consistently showed higher serum CAF22 levels than the healthy controls. However, the serum CAF22 levels were not correlated with the SPPB and SARC-F scores in individual cohorts of participants.

P3NP is a fragment of procollagen III and is released into the circulation during the final stages of collagen synthesis^22^. Higher circulating P3NP levels have been observed in degenerating conditions with generalized chronic inflammation and fibrosis^22^. An association between plasma P3NP and changes in lean muscle mass has been observed in hormonal therapies^23^ and exercise training^24^. Higher plasma P3NP levels have also been observed in sarcopenia, although the effect was more pronounced in women^23^. We report an additional increase in P3NP levels in advanced sarcopenia, eliciting the role(s) of increased chronic inflammation and fibrosis in COPD and CHF.

Osteonectin is a glycoprotein in the extracellular matrix and is involved in cell–matrix interaction. Overexpression of osteonectin inhibits differentiation of C_2_C_12_ muscle cells^25^. Accordingly, patients with myopathies show increased expressions of osteonectin^26^. There is also a correlation with aging as reduced muscle mass in sarcopenia is inversely related to the circulating osteonectin levels^27^. Our finding of higher osteonectin levels in age-associated comorbidities confirms and extends the usefulness of osteonectin as a biomarker of sarcopenia.

Irisin is a pro-myogenic factor and its serum expression increases following exercise^28^. Circulating irisin levels correlate well with biceps circumference^29^ and are also increased in mice with increased musculature due to myostatin deficiency^30^. Low circulating irisin levels in the elderly have emerged as a sensitive marker of muscle mass and strength in sarcopenia^31^. We report that the irisin concentration is further reduced in sarcopenia patients with COPD and CHF, eliciting its negative correlation with muscle mass and strength in the elderly. Importantly, exercise restores irisin levels and protects against muscle loss and lung dysfunction in COPD^32^.

FABP3 is highly expressed in skeletal muscle and works as a lipid chaperon, as consumption of high-fat diet increases its muscle expression^33^. FABP3 expression also increases in sarcopenic muscle and contributes to muscle atrophy and weakness by inducing endoplasmic reticulum stress^34^. We found higher levels of circulating FABP3 in the patient with COPD and CHF, which integrate with the contribution(s) of ER stress to skeletal muscle remodeling in COPD^35^ and CHF^36^.

MIF is a pro-inflammatory cytokine with implications in skeletal muscle atrophy and weakness in inflammatory myopathies^37^. Since sarcopenia is associated with high inflammation, we speculated that the circulating MIF levels may reflect the skeletal muscle weakness and atrophy in the elderly with varying degrees of sarcopenia. Accordingly, patients with COPD and CHF who had higher inflammatory cytokines and advanced sarcopenia, also expressed higher circulating levels of MIF.

Sarcopenia group in our cohorts include the elderly with reduced muscle mass, strength, and physical capacity. According to the revised definition by EWGSOP, low muscle strength has emerged as a key determinant of sarcopenia, while reduced muscle quantity is used for confirmation of sarcopenia^2^. These parameters along with reduced physical capacity determine the functional dependency in the elderly. Due to a lack of established circulating biomarkers of sarcopenia, our choice of biomarkers was based on our previous investigations and the published literature. The median cut-off was selected empirically to generate useful high- and low-risk groups. A significantly higher proportion of sarcopenic patients in the high-risk group shows the predictive accuracy of the biomarkers panel. Additionally, we show that the SARC-F questionnaire has acceptable diagnostic value and can be used as a fast and early assessment tool in sarcopenia.

From a practical standpoint, the battery of biomarkers can be useful in the screening of sarcopenia. These assays require ≤ 1 ml of plasma, which can be obtained from the blood drawn for other investigations. This ensures the screening without the need for additional blood sampling. Measurement and analysis of biomarkers can be performed in additional few hours to evaluate the diagnosis and/or risk probability of sarcopenia for each subject.

This study only incorporated male participants, so the inclusion of female participants to understand complex etiology of sarcopenia and the contribution of menopause to muscle loss may be required. Considering the multifactorial nature of sarcopenia, the evaluation of six biomarkers may not encompass the several molecular mechanisms dictating sarcopenia phenotype in the elderly. Thus, the use of additional biomarkers and optimizing the combinations of biomarkers is required to reflect the entire biological changes dictating skeletal muscle impairment in sarcopenia. Additionally, longitudinal studies are essential to evaluate the diagnostic and prognostic potential of biomarkers during therapeutic interventions. Since inflammation and oxidative stress are hallmarks of several aging-related diseases, muscle-specific biomarkers with minimal interference by other diseases is required to assess sarcopenia.

## Methods

### Study design and participants

We recruited 258 ambulatory participants as healthy controls (N = 87) and patients with COPD (N = 86) and CHF (N = 81) at the University of Health Sciences (UHS), Lahore, teaching hospital of Gomal Medical College (GMC), Dera Ismail Khan and cardiac rehabilitation center, Hayatabad Medical Complex (HMC), Peshawar, Pakistan. The healthy controls and COPD participants were taken from a large cohort of patients described elsewhere^9,10,16^. The ethical review committee at the UHS, Institutional ethics committee at GMC and the clinical ethics committee at the HMC approved this study. All participants were male, 55–74 years old and provided written informed consent. COPD was defined as FEV_1_%/forced vital capacity (FVC) < 0.7 with persistent respiratory symptoms according to the GOLD guidelines^38^. The inclusion criteria for chronic HF patients were a diagnosis of heart failure with left ventricular ejection fraction ≤ 40%. Subjects with stable phenotype were included while those with the unstable phenotype (infection, exacerbation and/or hospitalization in the past month), arthritis, myopathies, neurological diseases, unstable angina, major surgeries and prolonged bed rest within eight weeks of the visit to the clinics were excluded^39^. Subjects with higher plasma urea and/or creatinine were also excluded due to an independent association between plasma CAF22 levels and kidney function^40^. Hence, our study protocol is consistent with previous studies used to characterize muscle wasting in patients with COPD and CHF^41,42^. Based on the definition by the European Working Group on Sarcopenia in Older People (EWGSOP), sarcopenia was defined as low muscle strength (HGS < 27 kg), low muscle quantity (ASMI < 7 kg/m^2^) and low physical performance (SPPB ≤ 8 and/or gait speed ≤ 8 m/s)^2,43^. Data was collected from structural interviews, clinical examinations, laboratory investigations and measurements of physical parameters. SARC-F questionnaire was used as an independent and rapid diagnostic tool for sarcopenia^12^. The questionnaire investigates various measures of physical performance as each measure given a score from 0 to 2 with a maximal score of 10. A score ≥ 4 was taken as a predictor of sarcopenia. This study was conducted in accordance with the declaration of Helsinki^44^.

### HGS and body composition

HGS was measured by a digital handgrip dynamometer (CAMRY, South El Monte, CA, USA) as described before^10,16^. The participants were instructed to sit down with their elbows flexed at an angle of 90◦ with the dynamometer in hand in the supine position. The participants were then asked to squeeze the dynamometer with maximal strength in a smooth manner without rapid jerking or wrenching. No other body movement was allowed during the procedure. Three attempts were performed with each hand with 60-s rest between each attempt and the highest value was recorded for the analysis. Appendicular skeletal muscle mass (ASM) and fat mass were calculated with the bioelectrical impedance analysis scale (RENPHO, Dubai, UAE). ASM was divided by body area to get appendicular skeletal muscle mass index (ASMI), as described previously^16^.

### Measurement of physical performance

The physical performance was assessed by the SPPB score. This battery is composed of three timed tests: 4-m walking speed, balance, and chair-stand tests. Timed results from each test were rescored from zero (worst performers) to four (best performers). The sum of the results from the three categorized tests (ranging from 0 to 12) was used for the present analyses, as described elsewhere^45^.

The walking speed was evaluated by measuring the participant's usual gait-speed (in m/s) over a 4-m course (4-m walk test; 4MWT). Based on the sample population quartiles, the following cut-points were used to categories the gait speed: ≤ 0.38 m/s, a score of 1; 0.39–0.57 m/s, a score of 2; 0.58–0.76 m/s, a score of 3; ≥ 0.77 m/s, a score of 4^45^.

To assess the chair-stand test, the participants were asked to stand up from a chair with their arms folded across the chest five times in a row as quickly as possible. The time needed to complete the task was recorded. The quartiles for the length of the time required for this measure were used for scoring as follows: ≥ 17.0 s, a score of 1; 14.1–16.9 s, a score of 2; 11.9–14.0 s, a score of 3; and ≤ 11.8 s, a score of 4^45^.

To assess the balance test, the participants were asked to perform three increasingly challenging standing-positions: side-by-side position, semi-tandem position, and tandem position. Participants were asked to hold each position for 10 s. Participants were scored as 1 if they were able to hold a side-by-side standing position for 10 s, but were unable to hold a semi-tandem position for 10 s; a score of 2 if they were able to hold a semi-tandem position for 10 s, but were unable to hold a tandem position for more than 2 s; a score of 3 if they were able to stand in tandem position for 3–9 s; and a score of 4 if they were able to hold the tandem position for 10 s. Cut-off point for sarcopenia on SPPB score was taken as ≤ 8^2^.

### Measurement of circulating biomarkers

For analysis of circulating biomarkers, plasma samples were assayed using ELISA kits for P3NP (Cat # MBS9141338, MyBioSource, San Diego, USA), CAF22 (NTCAF, ELISA, Neurotune, Schlieren-Zurich, Switzerland), osteonectin (Cat # ab220654, Abcam, Abu Dhabi, UAE), irisin (Cat # MBS2600406, MyBioSource, San Diego, USA), FABP3 (Cat # ab243682, Abcam, Abu Dhabi, UAE) and MIF (Cat # ab100594, Abcam, Abu Dhabi, UAE) according to the manufacturer’s instructions.

### Measurements of plasma 8-isoprostanes, CRP and creatine kinase

We used ELISA to measure 8-isoprostanes (Cayman Chemical, Ann Arbor, MI, USA) and CRP (R&D Systems, Minneapolis, MN, USA) levels and biochemical assays to measure creatine kinase levels, as described previously^9^.

### Quantification of RNA using real time-PCR

Total RNA was extracted from the plasma using TRIzol reagent and the cDNA was prepared from 1 mg of the total RNA using a cDNA Synthesis kit (Bio-Rad, Hercules, CA, USA) as described previously^46^. 2.5 ng of cDNA samples were amplified using specific primers along with fast SYBR green master mix (Applied Biosystems, Grand Island, NY, USA). GAPDH rRNA was used as internal control. The data were analyzed using the ΔΔCt method.

### Spirometry and pulse oximetry

The FEV1 and FVC were measured using a portable spirometer (Contec SP10, China), according to standards set by the American Thoracic Society^47^. A commercially available pulse oximeter was used to measure SpO_2_ in healthy controls and patients with respiratory diseases (Nellcor N-200, Hayward, California).

### Statistical analysis

Anthropometric measurements of the participants were presented using mean and standard deviation as data met the assumption for normality. Analysis of variance was used to compare groups and Pearson correlation was employed to determine the strength of the relationship between individual cohorts and various physical and biochemical parameters. Simple logistic regression analysis was used to calculate the predicted probability scores for each circulating biomarker. The areas under curve (AUC) were calculated using receiver operating characteristics (ROC) analysis to test the utility of risk scores. Two-sample t-test for percent was used to compare SPPB and SARC-F scores among the groups. A *p* value < 0.05 was statistically significant.

## Supplementary Information

Supplementary Figure
