# Supplementary material for: Prediction of sarcopenia using a battery of circulating biomarkers
Source: Sci Rep. 2021 Apr 21;11:8632. doi: 10.1038/s41598-021-87974-6 (PMC8060253; doi:10.1038/s41598-021-87974-6)
Supplement: Supplementary file 1 — Supplementary Figure [file 41598_2021_87974_MOESM1_ESM.docx]

**Prediction of sarcopenia using a battery of circulating biomarkers**

**Rizwan Qaisar^1^, Asima Karim^1, 2^, Tahir Muhammad^3^, Islam Shah^4^, Javaidullah Khan^5^**

**^1^Basic Medical Sciences, College of Medicine, University of Sharjah, Sharjah, United Arab Emirates**

**^2^University of Health Sciences, Lahore, Pakistan**

**^3^Departmenr of Biochemistry, Gomal Medical College, Dera Ismail Khan, Pakistan**

**^4^Department of Cardiology, Al Qassimi Hospital, Sharjah, United Arab Emirates**

**^4^Department of Cardiology, Post Graduate Medical Institute, Hayatabad Medical Complex, Peshawar, Pakistan**

**Corresponding author**

**Rizwan Qaisar, MBBS, PhD**

***Department of Basic Medical Sciences***

***College of Medicine***

***University of Sharjah, Sharjah, UAE***

***Phone: +971 6 505 7254***

***Fax: +9716 5585 879***

***ORCID ID: 0000-0001-8485-7172***

***E-mail:*** [***rqaisar@sharjah.ac.ae***](mailto:amoselhi@sharjah.ac.ae)

**Supplementary Fig 1 (A - B) legend.** Plasma mRNA expressions of the markers of inflammation (A) and oxidative stress (B) in healthy controls and patients with COPD and CHF. Values are expressed as mean ± SEM, *p < 0.05. (Interleukin-10, IL-10; Transforming growth factor-beta 1, TGF-b1; c-c motif chemokine receptor 5, CCR5; c-x-c motif chemokine ligand 8, CXCL-8; Interleukin-6, IL-6; c-x-c motif chemokine ligand 2, CXCL2; adrenomedullin, ADM; superoxide dismutase-1, SOD1; glutathione synthetase, GSS; glutathione peroxidase-1, GPX1).


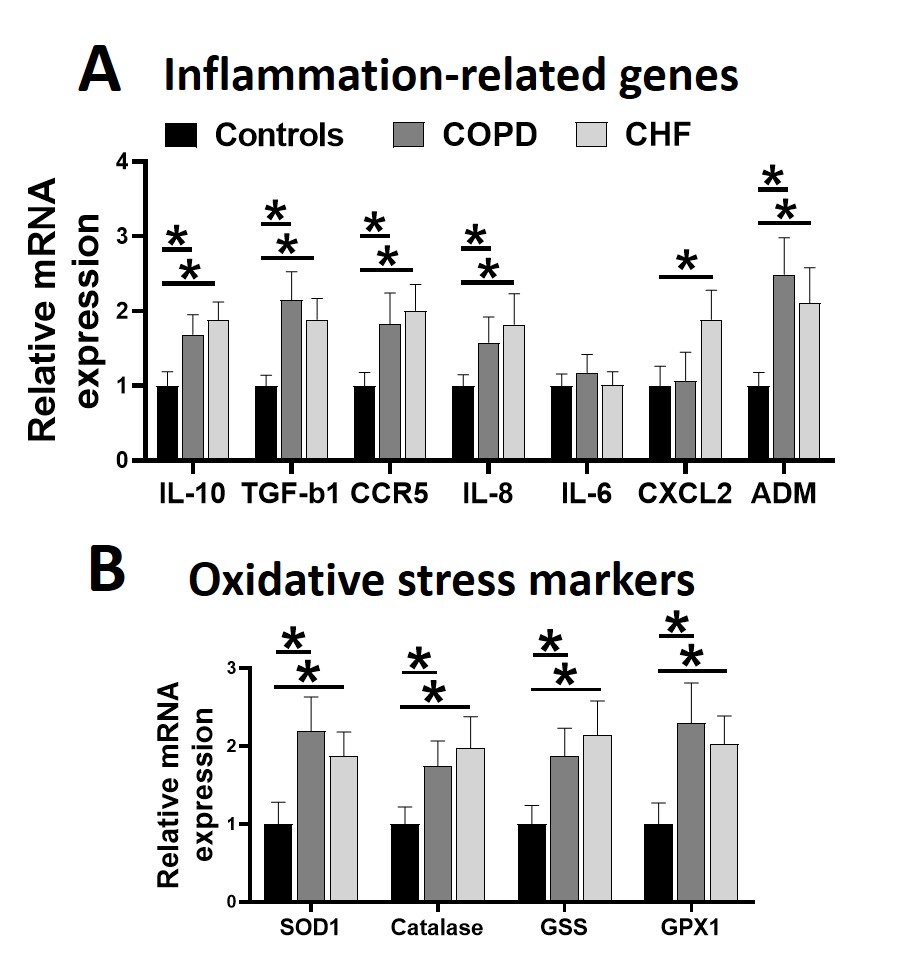


**Supplementary table 1.** List of primer sequences used for RT-PCR analysis. (Interleukin-10, IL-10; Transforming growth factor-beta 1, TGF-b1; c-c motif chemokine receptor 5, CCR5; c-x-c motif chemokine ligand 8, CXCL-8; Interleukin-6, IL-6; c-x-c motif chemokine ligand 2, CXCL2; adrenomedullin, ADM; superoxide dismutase-1, SOD1; glutathione synthetase, GSS; glutathione peroxidase-1, GPX1).

| **Gene name** | **NCBI Gene ID** | **Forward** | **Reverse** |
| --- | --- | --- | --- |
| **IL-10** | **3586** | TCTCCGAGATGCCTTCAGCAGA | TCAGACAAGGCTTGGCAACCCA |
| **TGF-b1** | **7040** | TACCTGAACCCGTGTTGCTCTC | GTTGCTGAGGTATCGCCAGGAA |
| **CCR5** | **1234** | TCTCTTCTGGGCTCCCTACAAC | CCAAGAGTCTCTGTCACCTGCA |
| **IL-8** | **3576** | GAGAGTGATTGAGAGTGGACCAC | CACAACCCTCTGCACCCAGTTT |
| **IL-6** | **3569** | AGACAGCCACTCACCTCTTCAG | TTCTGCCAGTGCCTCTTTGCTG |
| **CXCL2** | **2920** | GGCAGAAAGCTTGTCTCAACCC | CTCCTTCAGGAACAGCCACCAA |
| **ADM** | **133** | AAGAAGTGGAATAAGTGGGCT | TGTGAACTGGTAGATCTGGT |
| **SOD1** | **6647** | CTCACTCTCAGGAGACCATTGC | CCACAAGCCAAACGACTTCCAG |
| **Catalase** | **847** | GTGCGGAGATTCAACACTGCCA | CGGCAATGTTCTCACACAGACG |
| **GSS** | **2937** | AGCCAATGCTCTGGTGCTAC | ACCTTCGACGGATTACATGG |
| **GPX1** | **2876** | GTGCTCGGCTTCCCGTGCAAC | CTCGAAGAGCATGAAGTTGGGC |
| **GAPDH** | **2597** | GTCTCCTCTGACTTCAACAGCG | ACCACCCTGTTGCTGTAGCCAA |
